# Safety and efficacy of magnesium-rich artificial cerebrospinal fluid for subarachnoid hemorrhage

**DOI:** 10.3389/fneur.2024.1376216

**Published:** 2024-03-28

**Authors:** Yawen Cheng, Xiangning Han, Wanfu Xie, Gaofeng Xu, Xiaobin Bai, Lei Qi, Linjuan Zhang, Rui Liu, Weihua Dong, Weiyi Feng, Chengsen Pang, Wei Zhang, Fude Liu, Xiangqi Cao, Yue Xu, Guogang Luo

**Affiliations:** ^1^Department of Neurology and Stroke Center, The First Affiliated Hospital of Xi'an Jiaotong University, Xi'an, China; ^2^Department of Neurosurgery, The First Affiliated Hospital of Xi'an Jiaotong University, Xi'an, China; ^3^Department of Anesthesiology and Perioperative Medicine, The First Affiliated Hospital of Xi'an Jiaotong University, Xi'an, China; ^4^Department of Pharmacy Intravenous Admixture Services, The First Affiliated Hospital of Xi'an Jiaotong University, Xi'an, China

**Keywords:** aneurysmal subarachnoid hemorrhage, artificial cerebrospinal fluid, cerebral vasospasm, magnesium, prognosis

## Abstract

**Objectives:**

This study aimed to investigate the efficacy of using a newly formulated magnesium-rich artificial cerebrospinal fluid (MACSF) as an alternative to normal saline (NS) for intraoperative irrigation during aneurysm clipping in improving the prognosis of patients with Aneurysmal subarachnoid hemorrhage (aSAH).

**Methods:**

Patients with aSAH who underwent intraoperative irrigation with MACSF or NS during the clipping in the First Affiliated Hospital of Xi ‘an Jiaotong University from March 2019 to March 2022 were selected as MACSF group and NS group, respectively. The primary prognostic indicators were the incidence of favorable outcomes (mRS 0–2). The secondary outcome measures included cerebral vasospasm (CVS), mortality, total hospital stay, and intensive care unit (ICU) stay. Safety was evaluated based on the occurrence rates of hypermagnesemia, meningitis, and hydrocephalus.

**Results:**

Overall, 34 and 37 patients were enrolled in the MACSF and NS groups, respectively. At 90 days after aSAH onset, the proportion of favorable prognosis in the MACSF group was significantly higher than that in the NS group (*p* = 0.035). The incidence of CVS within 14 days after surgery was significantly lower in the MACSF group than that in the NS group (*p* = 0.026). The mortality rate in the MACSF group was significantly lower than in the NS group (*p* = 0.048). The median lengths of hospital stay (*p* = 0.008) and ICU stay (*p* = 0.018) were significantly shorter in the MACSF group than in the NS group. No significant differences were observed in safety measures.

**Conclusion:**

Using MACSF as an irrigation fluid for aneurysm clipping can significantly improve the 90-day prognosis of patients with aSAH, which may be related to the reduced incidence of CVS.

**Clinical trial registration:**

https://www.clinicaltrials.gov, identifier NCT04358445.

## 1 Introduction

Aneurysmal subarachnoid hemorrhage (aSAH) is a kind of stroke with high morbidity and mortality. Cerebral vasospasm (CVS) is a common complication of aSAH and is closely associated with patient death and disability of the patients (1, 2). Blood and its metabolites, being initiators of CVS, should be removed as soon as possible as they are initiators of CVS. Normal saline (NS), the most commonly used irrigation fluid in aneurysm clipping, can cause secondary brain injuries because its chemical properties of ion composition, osmolarity, and potential of hydrogen (pH) differ from those of cerebrospinal fluid (CSF) (3). Artificial cerebrospinal fluid (ACSF) has similar ionic salts, pH, and osmolality concentrations as CSF. Its safety and efficacy in neural tissues have been demonstrated (4–6). Using ACSF to flush nervous tissues did not alter the cerebrovascular reactivity or physicochemical properties of the CSF (3).

Magnesium (Mg), a vasodilator and natural calcium channel blocker, exerts unique protective functions in the brain. Intravenous magnesium administration is a potential therapeutic strategy for CVS. However, two recent randomized controlled trials (IMASH and MASH-2) failed to confirm the effectiveness of Mg in improving the prognosis of patients with aSAH patients (7, 8). Theoretically, direct intracisternal administration of Mg solution may be more effective.

However, no study has investigated whether using ACSF containing Mg as a flushing fluid during surgery is advantageous for relieving CVS and improving prognosis in patients with aSAH. Therefore, we formulated a new type of ACSF, magnesium-rich artificial cerebrospinal fluid (MACSF), which resembles physiological CSF and is enriched in Mg (9). We have verified that MACSF can maintain the normal physiological activity of rat basilar arteries *in vitro* and alleviate arterial hyper-responsiveness (10). In this study, MACSF was used as the irrigation fluid during aneurysm clipping and was compared with NS. Therefore, its impact on the 90-day prognosis and the CVS within 14 days postoperatively of patients with aSAH can be clarified.

## 2 Materials and methods

### 2.1 Study design

This was a single-center, non-randomized, non-blinded, single-arm trial conducted at the First Affiliated Hospital of Xi’an Jiaotong University (registered on ClinicalTrials.gov with NCT 04358445 on March 16, 2020) and approved by the Ethics Committee of the hospital (approval NO. XJYFY-2019 N28). All participants provided informed consent to participate in the study and shared their clinical data. All study-related documents were securely stored in the research center, and research data were collected in a limited manner. The entire research process was supervised and monitored by the clinical research center of our hospital. The study followed the SPIRIT reporting guidelines (11).

### 2.2 Study population

We enrolled 71 eligible patients with aSAH between March 2020 and March 2022. The MACSF group comprised patients who received MACSF as an intraoperative irrigation fluid, while the NS group consisted of patients who underwent surgery with NS as the irrigation fluid and were enrolled before March 2020. Patients were required to meet the following inclusion criteria: (1) age between 18 and 80 years, (2) aSAH diagnosed by CTA or DSA, (3) admission within 72 h after symptom onset, (4) aneurysm clipped within 36 h after admission, and (5) provided written informed consent. To avoid the influence of the modified Rankin Scale (mRS) score before aSAH onset on the prognostic evaluation, we added additional exclusion criteria after registration. These exclusion criteria included severe craniocerebral trauma, mRS score > 2 before the onset of aSAH, or severe concomitant diseases.

### 2.3 MACSF preparation

MACSF was prepared by trained medical personnel from the Department of Pharmacy Intravenous Admixture Services (PIAS) on a laminar flow clean bench. MACSF consists of a finished intravenous medication consisting of 0.9% sodium chloride injection, sterilized water for injection, 10% potassium chloride injection, 25% magnesium sulfate injection, 5% sodium bicarbonate injection, and 5% glucose injection. Freshly prepared MACSF was promptly transferred to the operating room in a designated container through a specific channel. The entire procedure was strictly to aseptic principles. A comparison of the compositions and properties of NS, MACSF, other ACSFs, and physiological CSF is shown in Table 1 (9).

**Table 1:** Comparison of physiological CSF, ACSF, and NS.

Composition	Physiological CSF	MACSF*	Artcereb*	Uchida ACSF*	NS
Na^+^(mEq/l)	145.5	146.2	145	145.5	154
K^+^(mEq/l)	2.8	2.7	2.8	2.8	0
Mg^2+^(mEq/l)	2.2	4.2	2.2	2.2	0
Ca^2+^(mEq/l)	2.5	0	2.3	2.3	0
Cl^−^(mEq/l)	111.9	123	129	128.5	154
HCO_3_^−^(mEq/l)	23.1	23.2	23.1	23.1	0
Glucose(g/l)	0.61	0.75	0.61	0.61	0
pH	7.31	7.35	7.3	7.3	6.7

CSF, cerebrospinal fluid; ACSF, artificial cerebrospinal fluid; NS, normal saline.

MACSF* is magnesium-rich artificial cerebrospinal fluid.

Artcereb* is an ACSF product named Artcereb^R^ developed by a Japanese research team (5).

Uchida ACSF* is an ACSF developed in the Pharmaceutical Division of Keio University School of Medicine (6).

### 2.4 Study interventions

From March 2020 to March 2022, eligible patients were recruited into the MACSF group and received MACSF as an intraoperative irrigation fluid. From March 2019 to March 2020, patients with aSAH who used NS as an irrigation fluid in surgery met the aforementioned criteria, and had complete case data, were recruited in the NS group as historical controls. Patients in the MACSF group were irrigated with MACSF after opening the skull and cerebral dura mater, whereas patients in the NS group were irrigated with NS throughout the surgery. All the patients underwent surgery and were managed by the same neurosurgical team. The remaining treatments in both groups strictly adhered to the clinical guidelines.

### 2.5 Clinical assessments

Demographic data, including sex, age, smoking and alcohol consumption history, and medical history, were recorded in detail. Vital signs, Hunt-Hess Scale scores, modified Fisher grades, and World Federation of Neurosurgical Societies (WFNS) scores were evaluated by a neurologist at admission. Cranial computed tomography was performed on admission and the day after surgery. The blood flow velocity of the intracranial arteries was dynamically evaluated using TCD performed by an experienced technician to determine the occurrence and severity of CVS every alternate day until 14 days after surgery. The Acute Physiology and Chronic Health Evaluation II (APACHE II) score of 15 was used as the criteria for admission and discharge from the ICU. Prognosis was assessed 90 days after disease onset using the mRS (12–14). All TCD examinations and clinical assessments were performed free of charge.

### 2.6 Evaluation of prognosis

Initially, we compared the mRS scores between the MACSF and NS groups at 1, 3, and 6 months after aSAH onset. Considering the representativeness and universality of the 90-day mRS evaluation in short-term prognosis, combined with the results of this study, we only chose 90 days as the final time point for prognostic evaluation. The prognosis was evaluated 90 days after ictus using the mRS, a 7-point scale ranging from 0 (no symptoms) to 6 (death). The mRS was dichotomized into favorable (mRS ≤ 2) and unfavorable (mRS > 2) prognosis (12, 15). Follow-up was conducted via telephone interviews by a trained neurologist blinded to the treatment assignments. If a patient was unavailable, a proxy was interviewed (16).

### 2.7 Definition of cerebral vasospasm

Ideally, cerebral angiography should be used as a diagnostic criterion for cerebral vasospasm. However, based on practical feasibility considerations, CVS was diagnosed using TCD in this study (17). The Lindeggard Index (LI) and mean blood flow velocity (MBF) are the main parameters used to evaluate the occurrence and severity of CVS. However, since some physiological or pathological conditions can also cause the increase of MBF without vasospasm, we eliminated the diagnostic criteria of “MBF of tested arteries is higher than 120 cm/s” to reduce the false-positive rate in diagnosing CVS. Therefore, the final criteria for diagnosing CVS by TCD are as follows (18, 19): (1) LI ≥ 3 or (2) Increase in MBF of tested arteries by more than 15 cm/s or 20% compared with the previous time. The severity of CVS is briefly described as follows (20–22): If the LI is greater than 6, severe CVS can be directly diagnosed. When LI ranges from 3 to 6, the severity of CVS can be classified by MBF as mild (120–139 cm/s), moderate (140–199 cm/s) or severe (≥200 cm/s).

### 2.8 Study end points

The primary outcome measures included the incidence of CVS within 14 days after surgery and the mRS score at 90 days after onset. Secondary prognostic indicators included length of total hospital stay, length of intensive care unit (ICU) stay, and mortality within 90 days of onset. The safety index was defined as the incidence of hypermagnesemia, meningitis, or hydrocephalus within 14 days after surgery.

### 2.9 Statistical analysis

According to previous epidemiological investigations, the incidence of CVS in the NS group was 60% (1, 2). Owing to the lack of studies related to ACSF, we hypothesized that using MACSF for intraoperative irrigation during clipping would reduce the incidence of CVS by 50%. Based on this assumption, with a two-sided significance level of 0.05 and power of 90%, at least 27 patients were recruited into the MACSF group. Furthermore, 33 patients were required when the loss-to-follow-up rate was empirically assumed to be 20%. However, we set the ratio of the control group to the experimental group at more than 1:1.

All statistical analyses were performed using SPSS 23.0. Data are presented as mean ± standard deviation ( $\bar{X}$ ± s) for continuous symmetric distribution variables, median (M) and interquartile range (IQR) for continuous skewed distribution variables, and percentages for categorical variables. Group comparisons were performed using Analysis of Variance or independent Student’s *t*-test for continuous variables and chi-squared test for categorical variables. The Mann–Whitney U non-parametric test was used for variables that did not meet the conditions of the parameter test. *p* < 0.05 was considered statistically significant.

### 2.10 Data availability statement

Data were recorded and stored using both paper forms and electronic databases. Supporting data for the findings of this study are available from the corresponding author upon request.

## 3 Results

Between March 2019 and March 2022, 71 eligible patients were enrolled (34 and 37 in the MACSF and NS groups, respectively). All patients completed the follow-up 90 days after onset. There were no significant differences between the two groups regarding age, sex, smoking and drinking history, comorbidities, family history of SAH, Hunt-Hess grade, modified Fisher grade, WFNS classification, or aneurysm characteristics. Vital signs and laboratory test results at admission were also included as baseline metrics, and there were no significant differences in these data between the two groups (Table 2).

**Table 2:** Comparison of baseline data in patients using MACSF* and NS* as irrigating fluid during the surgery of aneurysm clipping.

Variable	MACSF group *N* = 34	NS group *N* = 37	*p*
Age (y)	55.9 ± 8.4	58.7 ± 8.9	0.17
Female sex (*n*, %)	15 (44.1%)	23 (62.2%)	0.13
Medical history (*n*, %)			
Smoking	11 (32.4%)	12 (32.4%)	0.99
Drinking	7 (20.6%)	10 (27.0%)	0.53
Hypertension	20 (58.8%)	28 (75.7%)	0.13
Diabetes	2 (5.9%)	4 (10.8%)	0.68
Aneurysm	0 (0.0%)	1 (2.7%)	1.00
Hunt-Hess grade (*n*, %)			
I–II	9 (26.5%)	12 (32.4%)	0.58
III–V	25 (73.5%)	25 (67.6%)	
m-Fisher grade (*n*, %)			
0–2	19 (55.9%)	16 (43.2%)	0.29
3–4	15 (44.1%)	21 (56.8%)	
WFNS classification (*n*, %)			
I-II	22 (64.7%)	18 (48.7%)	0.17
III-V	12 (35.3%)	19 (51.3%)	
Aneurysmal Location (*n*, %)			
Anterior circulation	33 (97.1%)	35 (94.6%)	1.00
Posterior circulation	1 (2.9%)	2 (5.4%)	
Aneurysmal Number (*n*, %)			
Single	31 (91.2%)	31 (83.8%)	0.48
multiple	3 (8.8%)	6 (16.2%)	
Aneurysmal Size (*n*, %)			
≤5 mm	20 (58.8%)	15 (40.5%)	0.12
>5 mm	14 (41.2%)	22 (59.5%)	
Heart rate (bpm)	76.4 ± 11.6	79.8 ± 12.5	0.24
Breath (bpm)	17.6 ± 2.7	18.0 ± 2.9	0.57
Temperature (°C)	36.5 (36.3, 36.7)	36.5 (36.3, 36.7)	0.51
SBP (mmHg)	140.5 ± 15.8	136.9 ± 16.4	0.35
DBP (mmHg)	82.5 (78.8, 92.0)	80.0 (78.0, 89.5)	0.21
MAP (mmHg)	103.5 (94.5, 113.0)	99.3 (93.3, 109.8)	0.27
Hb (g/L)	131.3 ± 25.7	134.5 ± 19.9	0.56
MCHC (g/L)	335.5 (327.3, 342.0)	340.0 (330.0, 347.5)	0.10
RBC (×10^12^/L)	4.3 ± 0.7	4.4 ± 0.6	0.61
WBC (×10^9^/L)	11.4 (7.8,13.1)	10.7 (8.9,14.2)	0.63
Neu (%)	85.9 (77.8,91.8)	90.1 (83.3,92.5)	0.07
PLT (×10^9^/L)	192.0 (163.5, 230.3)	201.0 (162.5, 256.5)	0.46
AST (U/L)	23.0 (19.8, 29.5)	24.0 (18.5, 32.0)	0.62
ALT (U/L)	24.0 (14.8, 29.3)	22.0 (14.5, 28.5)	0.73
ALB (g/L)	40.3 (37.5, 42.7)	41.3 (38.8, 43.9)	0.35
Glu (mmol/L)	6.8 (5.8, 7.6)	6.8 (6.0, 7.6)	0.80
PT (s)	12.9 ± 1.3	13.3 ± 1.0	0.12
APTT (s)	32.2 ± 5.6	31.4 ± 4.1	0.51
BUN (mmol/L)	4.5 (3.7, 5.7)	4.6 (3.6, 5.3)	0.95
Cr (umol/L)	45.5 (37.0, 54.5)	47.0 (37.5, 58.5)	0.67
Ca^2+^ (mmol/L)	2.3 ± 0.1	2.3 ± 0.2	0.25
Mg^2+^ (mmol/L)	0.9 ± 0.1	1.0 ± 0.1	0.20

MACSF, magnesium-rich artificial cerebrospinal fluid.

NS, 0.9% sodium chloride injection.

Hunt-Hess, Hunt-Hess Scale scores.

m-Fisher, modified Fisher grades.

WFNS, World Federation of Neurosurgical Societies scores.

### 3.1 Clinical outcomes

**Figure 1:**
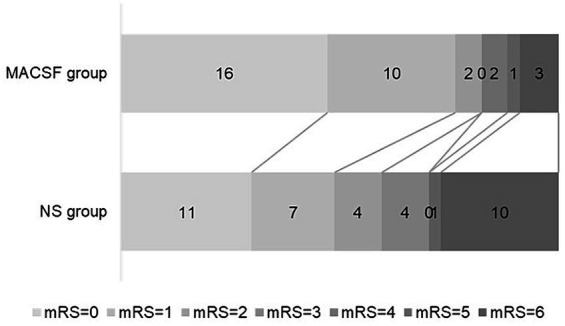
Outcome at 90-day after onset in patients using MACSF* and NS* as irrigating fluid during aneurysm clipping surgery. MACSF, magnesium-rich artificial cerebrospinal fluid. NS, 0.9% sodium chloride injection.

All patients completed 90-day follow-up examinations. The distribution of the mRS scores 90 days after onset is presented in Figure 1. A favorable outcome (mRS ≤ 2) at 90 days after onset was significantly more common in the MACSF group (82.35%) than in the NS group (59.46%; *p* = 0.035). There was also a significant difference in the mortality rate 90 days after onset between the two groups (8.82% vs. 27.03%; *p* = 0.048) (Table 3).

**Table 3:** Comparison of prognosis and mortality rate at 90 days after onset in patients using MACSF* and NS* as irrigating fluid during the surgery of aneurysm clipping.

Variable	MACSF group *N* = 34	NS group *N* = 37	*p*
mRS ≤ 2	28 (82.35%)	22 (59.46%)	**0.035**
mRS > 2	6 (17.65%)	15 (40.54%)	
mortality rate	3 (8.82%)	10 (27.03%)	**0.048**

MACSF, magnesium-rich artificial cerebrospinal fluid.

NS, 0.9% sodium chloride injection.Bold values are *p* < 0.05.

### 3.2 Cerebral vasospasm

**Figure 2:**
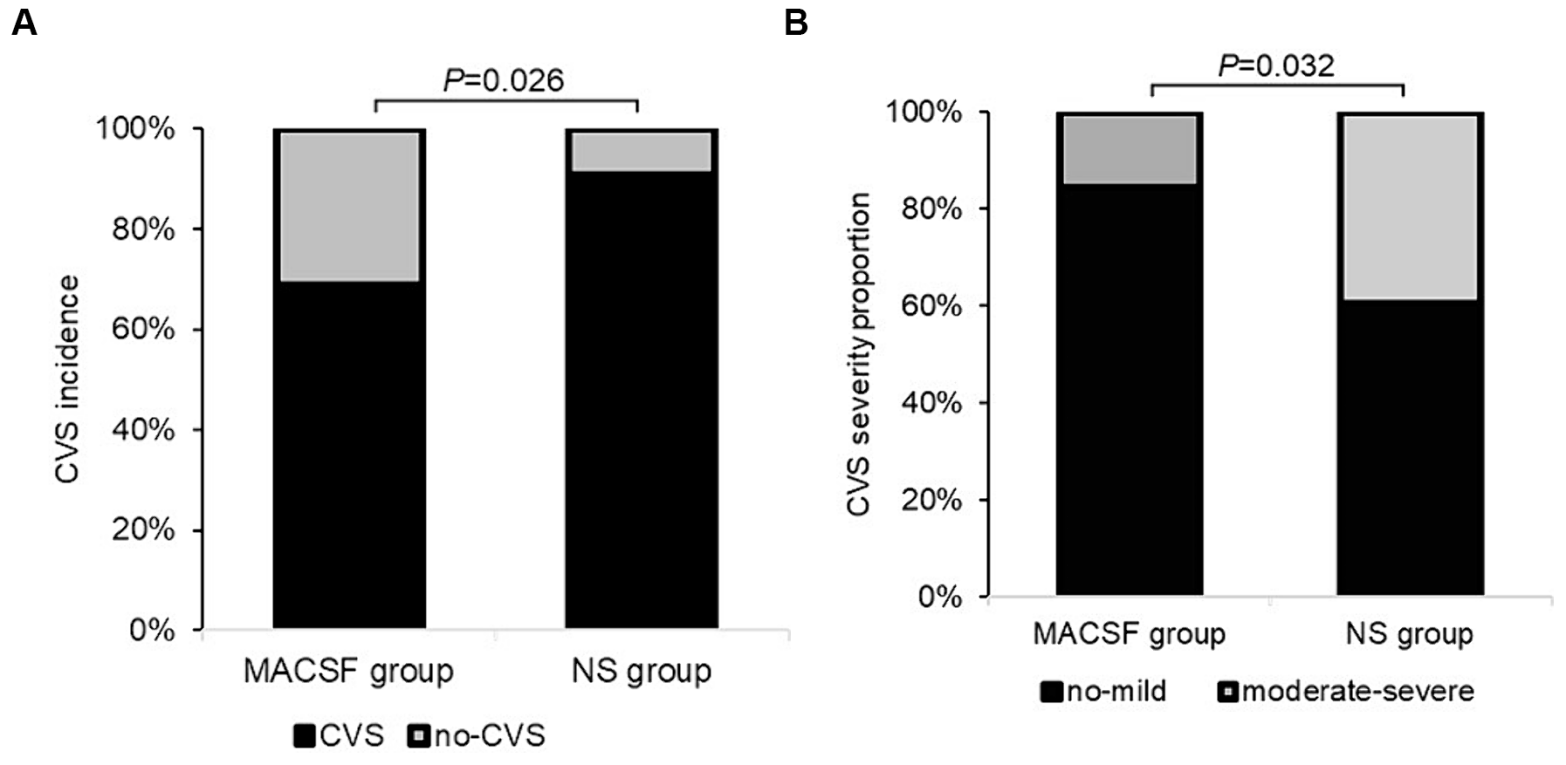
The incidence **(A)** and severity **(B)** of CVS within 14 days after the surgery of aneurysm clipping in patients with aSAH using MACSF* and NS* as intraoperative irrigation fluid. CVS, cerebral vasospasm. MACSF, magnesium-rich artificial cerebrospinal fluid. NS, 0.9% sodium chloride injection.

When analyzing the incidence of CVS, six patients were excluded because of TCD examinations less than 3 times (two cases in the MACSF group and four cases in the NS group). The overall incidence of CVS was 80.00%. There was no significant difference in the duration of anti-CVS drug use (days) between the MACSF (12.63 ± 6.54) and NS groups (14.15 ± 7.94; *p* = 0.402). The incidence and severity of CVS in the two groups are shown in Figure 2. Statistical analysis revealed that the incidence of CVS was significantly lower in the MACSF group (22 out of 32, 68.75%) than in the NS group (30 out of 33, 90.91%; *p* = 0.026), and the incidence of moderate-to-severe CVS was also significantly lower in the MACSF group (5 out of 32, 15.63%) than in the NS group (13 out of 33, 39.39%; *p* = 0.032).

### 3.3 Length of stay

**Figure 3:**
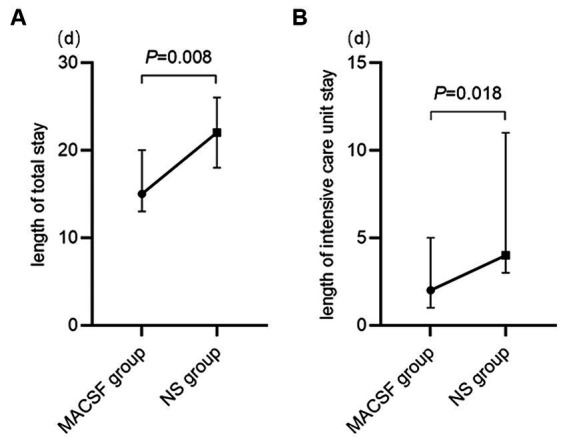
The length of total stay **(A)** and length of intensive care unit stay **(B)** of patients using MACSF* and NS* as irrigating fluid during the surgery of aneurysm clipping. MACSF: magnesium-rich artificial cerebrospinal fluid. NS, 0.9% sodium chloride injection.

Three and 10 patients in the MACSF and NS groups, respectively, were excluded because of voluntary discharge. As is shown in Figure 3, the median length of total hospital stay was significantly shorter in the MACSF group (M 15.00, IQR 11.00–22.00) than in the NS group (M 22.00, IQR 18.00–27.00; *p* = 0.008), and the median length of ICU stay was also significantly shorter in the MACSF group (M 2.00, IQR 0.00–5.00) than in the NS group (M 4.00, IQR 3.00–15.00; *p* = 0.018).

### 3.4 Serum Mg^2+^ and Ca^2+^ concentration

Serum Mg^2+^ and Ca^2+^ concentrations were measured on admission and within 24 h after surgery. In the MACSF group, the mean serum concentrations of Mg^2+^ and Ca^2+^ at admission were 0.93 ± 0.11 mmol/L and 2.29 ± 0.12 mmol/L, respectively. In the NS group, they were 0.96 ± 0.10 mmol/L and 2.33 ± 0.16 mmol/L. No significant differences were observed between the groups. The postoperative serum concentrations of Mg^2+^ and Ca^2+^ compared to those at admission were not significantly different in either the MACSF or NS groups.

### 3.5 Safety outcomes

The incidence of hypermagnesemia was not significantly different between the MACSF (0 out of 34, 0.00%) and NS groups (2 out of 37, 5.41%; *p* = 0.494). Additionally, the incidence of meningitis did not differ significantly between the two groups (MACSF group [15 out of 34, 44.12%] vs. NS group [16 out of 37, 43.24%]; *p* = 0.941). Furthermore, the difference in the incidence of hydrocephalus was not significant (MACSF group [3 out of 34, 8.82%] vs. NS group [2 out of 37, 5.41%]; *p* = 0.665) (Table 4).

**Table 4:** Comparison of complications in patients using MACSF* and NS* as irrigation fluid during the surgery of aneurysm clipping.

Complications	MACSF group *N* = 34	NS group *N* = 37	*p*
Hypermagnesemia	0 (0.00%)	2 (5.41%)	0.494
Meningitis	15 (44.12%)	16 (43.24%)	0.941
Hydrocephalus	3 (8.82%)	2 (5.41%)	0.665

MACSF, magnesium-rich artificial cerebrospinal fluid.

NS, 0.9% sodium chloride injection.

## 4 Discussion

This study confirmed that the use of MACSF as the irrigation fluid in aneurysm clipping surgery, which effectively reduced the incidence of CVS, improved the 90-day prognosis, and shortened the length of hospital stay in patients with aSAH.

MACSF is rich in magnesium ions, and irrigation with MACSF during aneurysm clamping can increase the concentration of Mg^2+^ in the CSF. As a natural antagonist of Ca^2+^, extracellular Mg^2+^ can reduce Ca^2+^–Na^+^ exchange on the cell membrane, preventing Ca^2+^ influx, vasoconstriction and CVS (23–25). Additionally, Mg^2+^ antagonizes the N-methyl-d-aspartate receptors in the brain, prevents glutamate stimulation, and reduces Ca^2+^ influx during ischemic injury (26, 27). Studies have demonstrated that Mg^2+^ is involved in the composition of various coenzymes and is related to cellular energy metabolism by reducing ATP consumption, damaging the cell membrane, and causing neuronal edema (25). However, two previous randomized controlled trials (IMASH and MASH-2) failed to confirm the effects of intravenous MgSO_4_ infusion on improving functional outcomes in patients with aSAH patients (7, 8). The most plausible reason for this outcome is that the increased serum Mg^2+^ concentration did not deliver an effective Mg^2+^ concentration in the CSF before inducing side effects. In our study, intraoperative irrigation with MACSF directly increased the concentration of Mg^2+^ in the CSF and better utilized the role of Mg^2+^ in combating CVS as well as protecting the neural tissue.

An animal study found that the CSF concentration of Mg^2+^ should be more than 3 mEq/L to dilate the spastic cerebral arteries in dogs effectively (28). It has been confirmed that continuous intracranial infusion of ACSF with an increased concentration of Mg^2+^ can cause vasodilation of the spastic cerebral arteries in dogs after SAH (29). Therefore, researchers recommend using ACSF with an appropriate concentration of Mg^2+^ as flushing fluid during neurosurgery to prevent CVS. Continuous intracisternal irrigation with an Mg-related solution appears to stabilize the CSF concentration of Mg^2+^ at an effective level to relieve CVS. One research team suggested that continuous intracisternal irrigation with 5 mmol/L (10 mEq/L) MgSO_4_ solution from days 4 to 14 after surgery could inhibit CVS in patients with aSAH (30). However, the overall prognosis did not improve. Furthermore, Mg-related side effects have been reported. Recently, it was demonstrated that intracisternal infusing with 2.5 mmol/L (5 mEq/L) MgSO_4_ solution, which was started immediately after surgery, could reduce the incidence of CVS and improve clinical outcomes in patients with poor-grade aSAH without Mg-related complications (31). This indicates that early elevation of the Mg^2+^ concentration in the CSF may be the key to alleviating CVS. However, continuous intracranial infusion has several disadvantages. This is traumatic and may increase the risk of meningitis. Furthermore, long-term bed rest after surgery increases the incidences of phlebothrombosis and pneumonia. In our study, we used MACSF only intraoperatively, which shortened the use time of the Mg solution and reduced the risk of complications.

Early intracranial perfusion with MACSF removes blood from the subarachnoid space, increases the CSF Mg^2+^ concentration, and inhibits the occurrence and development of CVS. CVS remission increases blood flow in affected arteries, improves oxygen supply to relevant brain tissues, maintains neurological function, and effectively improves patient prognosis. Intraoperative irrigation with MACSF, with an Mg^2+^ concentration of 4.2 mEq/L, did not increase the serum concentration of Mg^2+^ or cause Mg-related adverse reactions. Additionally, using MACSF alone in surgery does not increase pain and can avoid the complications of continuous intracisternal infusion for patients with aSAH. Since MACSF has not yet been industrialized, it was prepared by staff from the Department of PIAS, strictly following aseptic principles. Pollution during preparation and transportation was still possible; therefore, the incidence of meningitis was considered one of the indicators to evaluate safety. However, the current data indicates no significant differences in secondary infection rates between the two groups. Therefore, it is safe to use intravenous drugs to prepare MACSF in compliance with aseptic principles.

As a new type of ACSF that is easy to prepare and is widely used, MACSF may replace NS, Ringer’s solution, or other ACSF products, becoming a safe, effective, and convenient succedaneum for physiological CSF in the future.

But, this study adopted a historical control instead of a randomized control, which may have led to bias and a lower level of evidence than a standard randomized controlled trial (RCT). In the future, a planned RCT trial will validate the results of this study. The study sample was small because of the aSAH incidence and study period limitations (The epidemic period of COVID-19). Therefore, a larger study is needed to determine whether our protocol can be used as a standard therapeutic strategy. Lumbar puncture or lumbar cisternal drainage was not routinely performed after surgery; therefore, postoperative CSF samples were obtained from only a few patients. Consequently, the CSF concentrations of Ca^2+^, Mg^2+^ and spasmogens were not tested and should be assessed in the future.

All in all, our results suggest that using MACSF as an intraoperative irrigation fluid for aneurysm clipping can effectively reduce the incidence and severity of CVS, improve the prognosis 90 days after onset, and shorten the length of hospital stay without increasing the risk of complications in patients with aSAH. However, these findings need to be validated in randomized controlled trials.

## Data availability statement

The raw data supporting the conclusions of this article will be made available by the authors, without undue reservation.

## Ethics statement

The studies involving humans were approved by the Ethics Committee of the First Affiliated Hospital of Xi’an Jiaotong University. The studies were conducted in accordance with the local legislation and institutional requirements. The participants provided their written informed consent to participate in this study.

## Author contributions

YC: Conceptualization, Funding acquisition, Writing – original draft, Writing – review & editing. XH: Conceptualization, Visualization, Writing – original draft, Writing – review & editing. WX: Methodology, Resources, Writing – review & editing. GX: Methodology, Resources, Writing – review & editing. XB: Investigation, Writing – review & editing. LQ: Methodology, Supervision, Writing – review & editing. LZ: Supervision, Writing – review & editing. RL: Writing – review & editing, Methodology. WD: Writing – review & editing, Formal analysis. WF: Formal analysis, Writing – review & editing. CP: Writing – review & editing, Project administration. WZ: Writing – review & editing, Funding acquisition, Software. FL: Software, Writing – review & editing. XC: Writing – review & editing, Data curation. YX: Writing – review & editing, Formal analysis, Software. GL: Writing – review & editing, Conceptualization, Methodology, Project administration, Supervision, Writing – original draft.

## References

[1] Dorsch NW King MT. A review of cerebral vasospasm in aneurysmal subarachnoid haemorrhage part I: incidence and effects. J Clin Neurosci (1994) 1:19–26. doi: 10.1016/0967-5868(94)90005-1, PMID: 18638721

[2] Bacigaluppi S Zona G Secci F Spena G Mavilio N Brusa G. Diagnosis of cerebral vasospasm and risk of delayed cerebral ischemia related to aneurysmal subarachnoid haemorrhage: an overview of available tools. Neurosurg Rev (2015) 38:603–18. doi: 10.1007/s10143-015-0617-3 25732522

[3] Kazim SF Enam SA Shamim MS. Possible detrimental effects of neurosurgical irrigation fluids on neural tissue: an evidence based analysis of various irrigants used in contemporary neurosurgical practice. Int J Surg (2010) 8:586–90. doi: 10.1016/j.ijsu.2010.07.292, PMID: 20673818

[4] Elliott KAC Jasper HH. Physiological salt solutions for brain surgery—stuides of local PH and pial vessel reactions to buffered and unbuffered isotonic solutions. J Neurosurg (1949) 6:140–52. doi: 10.3171/jns.1949.6.2.0140 18124751

[5] Morioka Y Nishimura M Takehara H Doi K Naito S Yamauchi A. Intrathecal disposition of ARTCEREB (R) irrigation and perfusion solution for cerebrospinal surgery in rats. Biol Pharm Bull (2011) 34:688–92. doi: 10.1248/bpb.34.688, PMID: 21532158

[6] Milhorat TH Uchida K Yamada M Hayashi T Mine Y Kawase T. Possible harmful effects on central nervous system cells in the use of physiological saline as an irrigant during neurosurgical procedures. Surg Neurol (2004) 62:96–105. doi: 10.1016/j.surneu.2003.12.014, PMID: 15261494

[7] Wong GKC Chan MTV Boet R Poon WS Gin T. Intravenous magnesium sulfate after aneurysmal subarachnoid hemorrhage: a prospective randomized pilot study. J Neurosurg Anesthesiol (2006) 18:142–8. doi: 10.1097/00008506-200604000-00009, PMID: 16628069

[8] Dorhout Mees SM Algra A Vandertop WP van Kooten F Kuijsten HA Boiten J. Magnesium for aneurysmal subarachnoid haemorrhage (MASH-2): a randomized placebo-controlled trial. Lancet (2012) 380:44–9. doi: 10.1016/S0140-6736(12)60724-7, PMID: 22633825 PMC3391717

[9] Walton JN. Davson H-physiology of cerebrospinal fliud. Br Med J (1967) 3:356. doi: 10.1136/bmj.3.5561.356

[10] Cheng Y-W Guo Y-C Li G-L Deng YN Li WJ Xu GF. Effects of a new magnesium-rich artificial cerebrospinal fluid on contractile 5-hydroxytryptamine and endothelin receptors in rat cerebral arteries. Neurol Res (2019) 41:1015–23. doi: 10.1080/01616412.2019.1672383, PMID: 31559927

[11] Chan A-W Tetzlaff JM Gotzsche PC Altman DG Mann H Berlin JA. Explanation and elaboration: guidance for protocols of clinical trials. BMJ (2013) 2013:346. doi: 10.1136/bmj.e7586 PMC3541470 23303884

[12] van Swieten JC Koudstaal PJ Visser MC Schouten HJ van Gijn J. Interobserver agreement for the assessment of handicap in stroke patients. Stroke (1988) 19:604–7. doi: 10.1161/01.STR.19.5.604, PMID: 3363593

[13] Quinn TJ Dawson J Walters MR Lees KR. Functional outcome measures in contemporary stroke trials. Int J Stroke (2009) 4:200–5. doi: 10.1111/j.1747-4949.2009.00271.x 19659822

[14] Patel N Rao VA Heilman-Espinoza ER Lai R Quesada RA Flint AC. Simple and reliable determination of the modified Rankin scale score in neurosurgical and neurological patients: the mRS-9Q. Neurosurgery (2012) 71:971–5. doi: 10.1227/NEU.0b013e31826a8a56, PMID: 22843133

[15] Berkhemer OA. A randomized trial of intraarterial treatment for acute ischemic stroke. N Engl J Med (2015) 372:11–20. doi: 10.1056/NEJMoa1411587 25517348

[16] Ciccone A Valvassori L Nichelatti M Sgoifo A Ponzio M Sterzi R. Endovascular treatment for acute ischemic stroke. N Engl J Med (2013) 368:904–13. doi: 10.1056/NEJMoa1213701 23387822 PMC3708480

[17] Lysakowski C Walder B Costanza MC Tramèr MR. Transcranial Doppler versus angiography in patients with vasospasm due to a ruptured cerebral aneurysm—a systematic review. Stroke (2001) 32:2292–8. doi: 10.1161/hs1001.097108, PMID: 11588316

[18] Suarez JI Qureshi MI Yahia AB Parekh PD Tamargo RJ Williams MA. Symptomatic vasospasm diagnosis after subarachnoid hemorrhage: evaluation of transcranial Doppler ultrasound and cerebral angiography as related to compromised vascular distribution. Crit Care Med (2002) 30:1348–55. doi: 10.1097/00003246-200206000-00035, PMID: 12072693

[19] Li DD Chang JY Zhou CX Cui JB. Clinical diagnosis of cerebral vasospasm after subarachnoid hemorrhage by using transcranial Doppler sonography. Eur Rev Med Pharmacol Sci (2018) 22:2029–35. doi: 10.26355/eurrev_201804_14732, PMID: 29687859

[20] Lindegaard KF Nornes H Bakke SJ Sorteberg W Nakstad P. Cerebral vasospasm diagnosis by means of angiography and blood velocity measurements. Acta Neurochir (1989) 100:12–24. doi: 10.1007/BF01405268, PMID: 2683600

[21] Tsivgoulis G Alexandrov AV Sloan MA. Advances in transcranial doppler ultrasonography. Curr Neurol Neurosci Rep (2009) 9:46–54. doi: 10.1007/s11910-009-0008-7 19080753

[22] The Role of Transcranial Doppler in Cerebral Vasospasm: A Literature Review. 14th International Conference on Neurvascular Events after Subarachnoid Hemorrage (Vasospasm). CA: Los Angeles (2017).

[23] Chang JJ Mack WJ Saver JL Sanossian N. Magnesium: potential roles in neurovascular disease. Front Neurol (2014) 5:52. doi: 10.3389/fneur.2014.00052 24782823 PMC3995053

[24] Okada M Kaneko S. Pharmacological interactions between magnesium ion and adenosine on monoaminergic system in the central nervous system. Magnes Res (1998) 11:289–305. PMID: 9884987

[25] Regan RF Jasper E Guo YP Panter SS. The effect of magnesium on oxidative neuronal injury in vitro. J Neurochem (1998) 70:77–85. doi: 10.1046/j.1471-4159.1998.70010077.x, PMID: 9422349

[26] Murata T Dietrich HH Horiuchi T Hongo K Dacey RG Jr. Mechanisms of magnesium-induced vasodilation in cerebral penetrating arterioles. Neurosci Res (2016) 107:57–62. doi: 10.1016/j.neures.2015.12.005, PMID: 26712324 PMC4884497

[27] Wong GKC Chan MTV Poon WS Boet R Gin T. Magnesium therapy within 48 hours of an aneurysmal subarachnoid hemorrhage: neuro-panacea. Neurol Res (2006) 28:431–5. doi: 10.1179/016164106X115035, PMID: 16759446

[28] Mori K Yamamoto T Miyazaki M Hara Y Aiko Y Koike N. Optimal cerebrospinal fluid magnesium ion concentration for vasodilatory effect and duration after intracisternal injection of magnesium sulfate solution in a canine subarachnoid hemorrhage model. J Neurosurg (2011) 114:1168–75. doi: 10.3171/2010.10.JNS10866, PMID: 21073257

[29] MORI K YAMAMOTO T MIYAZAKI M HARA Y KOIKE N NAKAO Y. Potential risk of artificial cerebrospinal fluid solution without magnesium ion for cerebral irrigation and perfusion in neurosurgical practice. Neurol Med Chir (Tokyo) (2013) 53:596–600. doi: 10.2176/nmc.oa2012-0295, PMID: 24067770 PMC4508684

[30] Yamamoto T Mori K Esaki T Nakao Y Tokugawa J Watanabe M. Preventive effect of continuous cisternal irrigation with magnesium sulfate solution on angiographic cerebral vasospasms associated with aneurysmal subarachnoid hemorrhages: a randomized controlled trial. J Neurosurg (2016) 124:18–26. doi: 10.3171/2015.1.JNS142757, PMID: 26230471

[31] Takeuchi S Kumagai K Toyooka T Otani N Wada K Mori K. Intravenous hydrogen therapy with intracisternal magnesium sulfate infusion in severe aneurysmal subarachnoid hemorrhage. Stroke (2021) 52:20–7. doi: 10.1161/STROKEAHA.120.031260, PMID: 33349011

